# Évaluation du système de surveillance des fièvres hémorragiques virales dans la wilaya d’Assaba, Mauritanie (2020-2022)

**DOI:** 10.48327/mtsi.v4i2.2024.513

**Published:** 2024-05-02

**Authors:** Boushab Mohamed BOUSHAB, Pauline Kiswendsida YANOGO, Mohamedou Hmeied MAHAM, Herman YODA, Djibril BARRY, Ahmed EL-BARA, Nicolas MEDA

**Affiliations:** 1 Service de médecine interne et maladies infectieuses, Centre hospitalier de Kiffa, Assaba, Mauritanie; 2 Burkina Field Epidemiology and Laboratory Training Program. Université Joseph Ki-Zerbo, Ouagadougou, Burkina Faso; 3 Direction générale des services de santé des forces armées et de sécurité, Nouakchott, Mauritanie; 4 Institut national de recherche en santé publique, Nouakchott, Mauritanie

**Keywords:** Évaluation, Utilité globale, Simplicité, Flexibilité, Acceptabilité, Système, Fièvres hémorragiques virales, Surveillance intégrée des maladies et riposte, SIMR, Assaba, Mauritanie, Afrique subsaharienne, Evaluation, Global utility, Simplicity, Flexibility, Acceptability, System, Viral hemorrhagic fevers, Integrated Disease Surveillance and Response, IDSR, Assaba, Mauritania, Sub-Saharan Africa

## Abstract

**Introduction:**

La surveillance des fièvres hémorragiques virales en Mauritanie fait partie de la Surveillance intégrée des maladies et riposte (SIMR). Peu d’informations sont disponibles sur l’évaluation du système de surveillance des fièvres hémorragiques virales dans la wilaya (région) de l’Assaba en Mauritanie.

**Méthodes:**

Une étude transversale descriptive a été menée de juillet à août 2022 dans la wilaya de l’Assaba avec comme objectif d’évaluer les caractéristiques du système en interrogeant tous les acteurs impliqués dans la surveillance des fièvres hémorragiques virales dans la wilaya de l’Assaba, en mettant l’accent sur la fièvre de la Vallée du Rift et la fièvre hémorragique de Crimée-Congo à l’aide des questionnaires élaborés sur la base des directives des *Centers for Disease Control and Prevention.* La base de données de 2020-2022 des fièvres hémorragiques virales du laboratoire de l’Institut national de recherche en santé publique a été analysée. Les médianes, les intervalles interquartiles et les proportions ont été calculés à l’aide des logiciels Epi Info^®^ 7.2.5.0 et Excel^®^ 2021.

**Résultats:**

L’entretien a concerné 26 acteurs impliqués dans le système de surveillance des fièvres hémorragiques virales. La majorité des répondants de l’enquête ont trouvé le système utile (51 %), simple (63 %), acceptable (46 %), réactif (64 %) et flexible (46 %). Selon l’analyse de la base de données de l’Institut national de recherche en santé publique, les cas de fièvres hémorragiques virales ont été pris en charge rapidement. Les réponses à l’enquête et l’analyse de la base de données ont révélé l’existence de problèmes sur le plan de la qualité des données et des mécanismes de gestion des données.

**Conclusion:**

Le système de surveillance des fièvres hémorragiques virales dans la wilaya de l’Assaba est fidèle à l’organisation et au fonctionnement du système national de surveillance des fièvres hémorragiques virales qui fait partie de la SIMR. Les caractéristiques d’utilité, de simplicité, de réactivité et de flexibilité du système de surveillance des fièvres hémorragiques virales sont satisfaisantes, mais l’acceptabilité et la flexibilité doivent être améliorées.

## Abstract (en)

**Introduction:**

Several arboviral diseases have been known to be endemic (e.g., Crimean-Congo hemorrhagic fever, Rift Valley fever) or are emerging (dengue fever, chikungunya, O’nyong-nyong) in human populations in Mauritania, while others have become rare in recent years (e.g. yellow fever). Moreover, domestic animals, especially cattle, camels, goats, and sheep, are also known to be infected with some of these arboviruses (e.g. Crimean-Congo hemorrhagic fever, Rift Valley fever). For these reasons, viral hemorrhagic fever surveillance in Mauritania is part of the Integrated Disease Surveillance and Response (IDSR). However, limited information is available on the efficacy of the viral hemorrhagic fever surveillance system in the Assaba region of Mauritania. The aim of the present study was to assess the performance of the surveillance system, in particular its general utility, simplicity, flexibility, acceptability, and reactivity.

**Methods:**

A descriptive cross-sectional study was conducted from July to August 2022 in the Assaba region with the objective of evaluating the characteristics of the system by interviewing key actors involved in the surveillance of viral hemorrhagic fevers, with a focus on Rift Valley fever and Crimean-Congo hemorrhagic fever, using questionnaires developed following the guidelines of the Centers for Disease Control and Prevention (Atlanta, Georgia, USA). Data from 2020-2022 on viral hemorrhagic fevers from the National Institute of Public Health laboratory were analyzed. Medians, interquartile ranges, and proportions were calculated using Epi Info^®^ 7.2.5.0 and Excel^®^ 2021.

**Results:**

The questionnaire was answered by all twenty-six persons involved in the viral hemorrhagic fever surveillance system in Assaba region. The majority of survey respondents found the system to be useful (51%), simple (63%), acceptable (46%), responsive (64%), and flexible (46%). An analysis of the data revealed a positive predictive value of 28% for Rift Valley Fever. The weekly distribution of cases within the wilaya indicates that the moughataa of Kiffa recorded the highest number of cases in September, with a notable weekly peak during that month in 2020. According to the analysis of the National Institute of Public Health database, cases of viral hemorrhagic fevers were promptly handled. Survey responses and database analysis revealed issues related to data quality and data management mechanisms. These limitations in the surveillance system are likely to be due to insufficient resources and training of the personnel, in particular with regards to data collection and management, which in turn led to incomplete or missing data and invalid data entry. These weak points can be ascribed, at least in part, to financial constraints and inadequate attribution of priority to arboviral diseases. Despite these limitations, disease data generated by the surveillance system were generally reliable.

**Conclusion:**

The viral hemorrhagic fever surveillance system in the Assaba region adheres to the organization and functioning of the national viral hemorrhagic fever surveillance system, which is part of the IDSR. The characteristics of utility, simplicity, responsiveness, and flexibility of the viral hemorrhagic fever surveillance system are good, but acceptability and flexibility need further improvement. The earlier the first arboviral human or animal cases are detected, the more likely an active intervention can be organized in response to the emerging epidemic or epizootic and prevent the spread of the disease. An efficient viral surveillance system is the key to reducing the negative impact of arboviral diseases in Assaba region.

## Introduction

Les fièvres hémorragiques virales (FHV) sont des arboviroses qui associent à une fièvre généralement élevée des signes hémorragiques d’intensité variable avec un taux de létalité important [6]. Les virus responsables appartiennent à différentes familles dont les Flaviviridae (dengue, fièvre jaune, Omsk, Kyasanur, Alkhurma), les Bunyaviridae (fièvre hémorragique de Crimée-Congo (FHCC), fièvre de la vallée du Rift (FVR)) transmis par des moustiques ou des tiques, les Arenaviridae (Lassa, Junin), les Hantaviridae transmis par les déjections de rongeurs et les Filoviridae (Ebola, Marburg) pour lesquels le réservoir naturel reste mal connu [6, 25]. Les zones d’endémie des FHV se trouvent dans le monde entier, mais le diagnostic de confirmation d’une FHV est classiquement effectué dans de grands laboratoires de référence [25]. En Mauritanie, il existe deux types de FHV : la FHCC, découverte pour la première fois en 1983 [20], et la FVR en 1987 [3]. Depuis lors, le pays a connu plusieurs épidémies de FHV [3, 4, 5, 7, 8, 15, 17, 20, 21]. La surveillance des FHV en Mauritanie fait partie de la Surveillance intégrée des maladies et riposte (SIMR) [17].

Ces viroses, potentiellement graves, présentent des analogies dans la variabilité de leurs manifestations cliniques chez les patients [6, 9, 25]. Certaines particularités méritent néanmoins d’être mentionnées, telles que la prédominance des signes digestifs avec Ebola, l’atteinte ophtalmologique au cours de la FVR et la rhabdomyolyse au cours de la FHCC [25]. Généralement, les cas humains de FVR sont précédés ou concomitants d’une épizootie de FVR chez les ruminants (chèvres, moutons, chameaux) [3].

Le diagnostic et la prise en charge sont difficiles en raison de la non-spécificité des premiers symptômes, des installations de laboratoire limitées dans les zones endémiques, de la gravité de la maladie, du manque de traitement efficace, des exigences strictes en matière de contrôle des infections et de la propension à provoquer des épidémies avec des cas secondaires chez les travailleurs de la santé [9]. Dans de nombreux cas, des études détaillées n’ont été réalisées qu’après l’apparition d’épidémies et une surveillance active est alors nécessaire pour empêcher la dissémination virale dans les populations humaines [16]. Le système de surveillance des FHV est passif, les cas suspects étant identifiés dans les établissements de santé. Il est donc crucial de s’assurer de l’efficacité de notre système de surveillance des maladies à potentiel épidémique (MPE) comme les FHV. Les objectifs du système de surveillance des FHV sont de détecter les cas à un stade précoce pour une prise en charge et un contrôle rapide, et de surveiller le taux de morbidité et le schéma de propagation. Nous avons cherché à déterminer si les objectifs du système de surveillance sont atteints et à évaluer la performance des caractéristiques du système de surveillance des FHV.

## Matériels et méthodes

**Figure 1:**
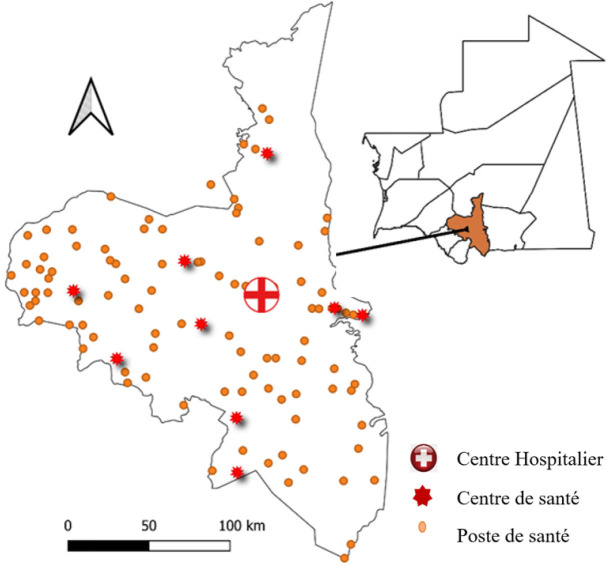
Cartographie sanitaire de la wilaya de l’Assaba. Source des données, ministère de la Santé Health mapping of the Assaba region. Data source: Ministry of Health

Notre étude s’est déroulée sur la wilaya (région) de l’Assaba, la 3^e^ région administrative du pays, couvrant une superficie de 36 600 km^2^ (Fig. 1). La population de cette région compte 326 000 habitants, représentant 9,2 % de la population nationale, avec une densité de population de 8,9 habitants/km^2^.

L’Assaba se caractérise par la présence d’une plaine alluviale sujette aux inondations pendant la saison des pluies, entravant ainsi la mobilité des habitants entre les localités et limitant l’accès aux services de santé. Plus de trois quarts de son territoire sont soumis à un climat saharien, caractérisé par des températures extrêmement élevées. Cette wilaya connaît une saison sèche, de novembre à juillet, et une saison des pluies, de juillet à octobre. Elle est subdivisée en cinq moughataas (départements) et compte 26 communes. Le système de santé en Mauritanie est structuré en trois niveaux : central, intermédiaire et régional, avec une organisation similaire dans l’Assaba. L’offre de soins suit la structure administrative du système de santé de la wilaya, avec le Centre hospitalier de Kiffa en tant que centre de référence. La surveillance épidémiologique, couvrant les FHV et d’autres MPE, est intégrée à tous les niveaux, avec une notification hebdomadaire des MPE et une compilation et analyse des données au niveau central. Notre étude, menée entre juillet et août 2022, est de nature transversale descriptive, utilisant une approche mixte (quantitative et qualitative). Elle a porté sur tous les cas de FHV enregistrés entre le 1^er^ janvier 2020 et 31 décembre 2022 dans le cadre de la surveillance épidémiologique.

## Définitions opérationnelles

### Organisation et fonctionnement

Dans la région d’Assaba, la surveillance des FHV s’intègre dans le cadre du dispositif de SIMR. Ce processus de surveillance des FHV est structuré en plusieurs niveaux opérationnels, avec une collaboration active des professionnels de santé locaux pour détecter précocement les cas et prendre des mesures appropriées. Lors de la confirmation des cas, une enquête multidisciplinaire est déclenchée, combinant différentes approches pour évaluer l’étendue de l’épidémie. Au niveau de la wilaya, la coordination des activités de surveillance est assurée par la Direction régionale de la santé (DRS). Les points focaux de surveillance au niveau moughataas et wilaya sont chargés de la compilation et de la transmission des données au niveau central, avec une périodicité journalière, hebdomadaire, mensuelle et trimestrielle. Les moyens de communications étaient la messagerie électronique, les appels ou messages téléphoniques ou les réseaux sociaux, notamment WhatsApp. Le niveau central est chargé de l’analyse et l’interprétation des données.

Les définitions des termes « utilité globale », « simplicité », « flexibilité », « acceptabilité » et « réactivité » utilisés dans ce travail sont donnés dans ce qui suit.

**Utilité globale:** Un système de surveillance est « globalement utile » pour prévenir et contrôler les événements sanitaires s’il améliore la compréhension de leurs impacts sur la santé publique. L’évaluation de l’utilité, selon les directives des Centres pour le contrôle et la prévention des maladies des États-Unis d’Amérique, soit *Centers for Disease Control and Prevention* (CDC), inclut l’examen du fonctionnement, des objectifs, et une analyse quantitative des données épidémiologiques. Les répondants partagent leur utilisation du système, leur avis sur les données des FHV et des suggestions pour une meilleure adaptation aux besoins [26]. Cet indicateur a été évalué en se basant sur la détection du nombre de cas par semaine épidémiologique, le taux d’attaque, la létalité, ainsi que la mise en œuvre de mesures de contrôle et de prévention par rapport au nombre total de critères évalués pour l’utilité globale.

**Simplicité:** La simplicité se réfère à la fois à la structure et à la facilité d’utilisation d’un système de surveillance. La simplicité est définie comme la facilité d’acheminement des données et de gestion du système [12]. Il est important que le système soit « simple » afin d’obtenir l’adhésion des acteurs responsables de la collecte des données et de s’assurer que le système de surveillance demeure peu contraignant à mettre en œuvre (par exemple, une quantité de données à collecter limitée au strict minimum essentiel, un formulaire facile à remplir en moins de 10 minutes, une faible périodicité de collecte des données, c’est-à-dire mensuelle). L’évaluation de ce paramètre s’est appuyée sur les réponses des intervenants dans l’enquête, portant sur la simplicité d’utilisation du système et sa fiabilité dans la collecte, la gestion et l’accès aux données. La simplicité a été évaluée en moyennant les notations attribuées par les intervenants pour l’adhésion et la fiabilité dans la collecte, la gestion et l’accès aux données.

**Flexibilité:** La flexibilité d’un système de surveillance est définie comme sa capacité de s’ajuster facilement aux évolutions des besoins d’information et des conditions opérationnelles, nécessitant peu d’investissements en temps, personnel ou fonds [13]. Un système de surveillance qui est flexible peut intégrer de nouvelles maladies, s’adapter aux problèmes de santé émergents, ajuster les définitions de cas et varier les sources de notification. Elle a été évaluée sur la base de l’intégration du système avec d’autres systèmes de surveillance et de l’adaptabilité aux besoins changeants du système [13]. Cet indicateur a été évalué en moyennant les notations attribuées par les intervenants pour les critères de la souplesse du système de surveillance.

**Acceptabilité:** C’est une mesure selon laquelle le personnel de surveillance est disposé à mettre en œuvre le système et les utilisateurs du système sont disposés à utiliser les données générées par le système [13]. L’acceptabilité a été évaluée au moyen des questions de l’enquête soumises aux intervenants portant sur les aspects du système favorisant ou entravant l’acceptabilité du système. Nous avons déterminé l’acceptabilité de la surveillance en nous basant sur l’exhaustivité et la rapidité de la notification [25]. Cet indicateur a été évalué en moyennant les notations attribuées par les intervenants pour la notification du système de surveillance.

**Réactivité** : Ce paramètre correspond à la rapidité de succession des diverses étapes d’un système de surveillance [13]. Le délai entre la date de l’épisode et celle de la déclaration au système a été examiné pour chaque cas afin d’évaluer la réactivité. Cette dernière a été mesurée par le nombre de sites de notification signalant des cas et le temps nécessaire aux DRS pour recevoir des informations des établissements de santé en cas de suspicion de cas [14].

La valeur prédictive positive (VPP) est la proportion de personnes identifiées comme ayant effectivement la maladie sous surveillance [13]. Le questionnaire utilisé pour évaluer le fonctionnement du système de surveillance, c’est-à-dire l’utilité globale, la simplicité, la flexibilité, l’acceptabilité et la réactivité, est présenté en Annexe.

### Définitions des cas

**Cas suspect:** Tout cas de fièvre élevée à début brutal, ne répondant pas aux médicaments antipaludiques appropriés, accompagnée d’une hémorragie (épistaxis, hémoptysie, méléna, hématémèse, gingivorragie, ecchymose) ou de méningo-encéphalite ou ayant un lien épidémiologique avec des cas confirmés ou une flambée épidémique.

**Cas confirmé:** Cas suspect confirmé en laboratoire (sérologie positive des immunoglobulines M (IgM), transcription inverse-réaction en chaîne par polymérase (RT-PCR) positive, isolement du virus ou séroconversion IgM/immunoglobulines G (IgG) par enzyme-linked immunosorbent assay [ELISA]).

**Cas probable:** Tout cas suspect décédé dans un contexte hémorragique avant que l’on procède à la confirmation biologique.

**Cas contact:** Toute personne vivant au contact d’un cas confirmé, suspect ou probable.

## Collecte des données

Les personnes impliquées dans la surveillance des FHV à tous les niveaux de la pyramide sanitaire ont constitué la population enquêtée. Au niveau des formations sanitaires périphériques, les unités de surveillance épidémiologique, un échantillonnage aléatoire a été réalisé pour sélectionner les répondants dans la wilaya.

Au niveau intermédiaire, le centre hospitalier régional de Kiffa, qui est la structure de référence de la région, a aussi été inclus.

Au niveau central, ont été inclus la Direction générale de la santé publique (DGSP), la Direction de l’information stratégique et de la surveillance épidémiologique (DISSE) et l’Institut national de recherche en santé publique (INRSP).

L’interview des répondants s’est fait à l’aide d’un questionnaire et le choix de ces répondants s’est basé sur leur implication dans la surveillance des FHV. Au total, 26 personnes ont été concernées, soit une personne par unité de surveillance.

Nous avons examiné le manuel de surveillance intégrée des maladies et des réponses en Mauritanie et extrait des données de la période entre le 1^er^ janvier 2020 et 31 décembre 2022 pour les FHV à partir de la base de données commune des FHV (FVR, FHCC et dengue) du laboratoire de INRSP en Mauritanie, en utilisant un fichier Excel^®^ de 2021. Pour cette étude, nous avons fait appel au guide d’évaluation des systèmes de surveillance du CDC afin d’évaluer la performance du système de surveillance [12].

### Variables d’étude

Nous avons examiné les variables suivantes: l’âge, le genre et le lieu de résidence (moughataa) des patients, la date de notification des cas, la date d’investigation et de prélèvement des échantillons, la date de réception des échantillons au laboratoire, la date de confirmation des cas par le laboratoire de l’INRSP ainsi que la date de communication de ces informations.

### Analyse et traitement des données

Nous avons effectué une analyse descriptive des données à l’aide du logiciel Epi Info^®^ version 7.2.5.0. Les doublons ont été supprimés et les données manquantes ont été complétées à partir des registres disponibles à l’INRSP. Les caractéristiques ont été appréciées par l’estimation de la moyenne des résultats obtenus sur chaque variable.

## Résultats

Le système de surveillance des FHV a été soumis à une évaluation complète avec une participation de tout le personnel (n = 26; 100 %) impliqué dans la wilaya d’Assaba. Parmi les 26 individus interrogés, on compte 11 médecins (42 %), 14 infirmiers (54 %) et un biologiste (4 %). Tous les intervenants impliqués dans la surveillance ont affirmé que les FHV sont officiellement classées comme maladies à déclaration obligatoire (MDO) et qu’une définition de cas est actuellement en place.

**Figure 2:**
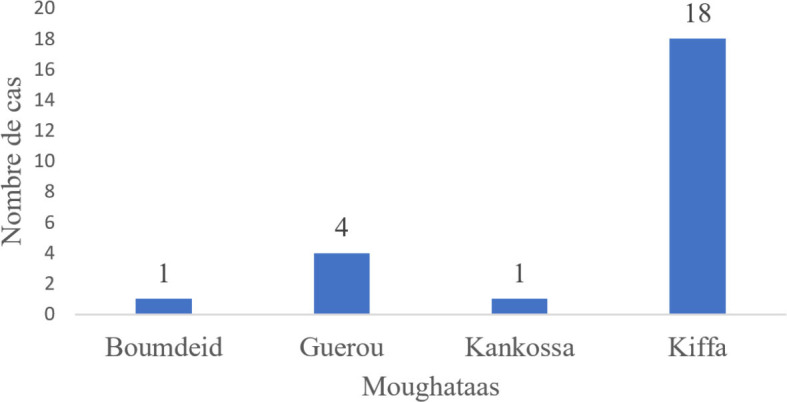
Répartition des cas de FHV de 2020 à 2022 selon les moughataas Distribution of FVH cases from 2020 to 2022 by moughataas

**Figure 3:**
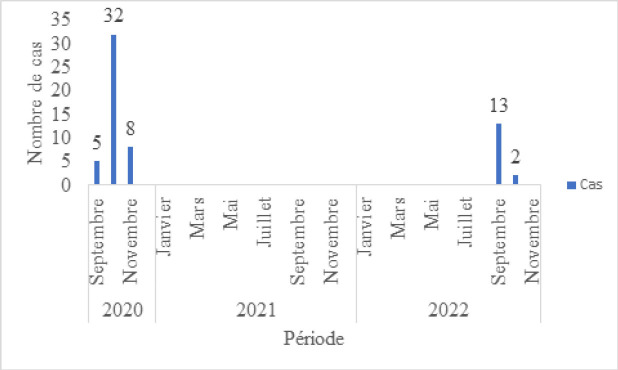
Repartions des cas de FHV selon la période de 2020 à 2022 Distribution of FHV cases by period from 2020 to 2022

Concernant les résultats de l’évaluation, un niveau moyen d’utilité de 51 % a été relevé, avec un intervalle de confiance (IC) à 95 % [16 - 85 %]. La simplicité moyenne était de 63 %, IC [33- 94 %]. Le niveau de performance en termes de flexibilité s’élevait à 46 %, IC [16-77 %]. De même, le niveau de performance en ce qui concerne l’acceptabilité était de 46 %, IC [21 - 70 %]. La réactivité moyenne était de 64 %, IC [39 - 89 %] (Tableau I). Une analyse des données de l’INRSP pour la période de 2020 à 2022 a révélé une valeur prédictive positive (VPP) de 28 *%* pour la FVR (Tableau II), avec un IC [13- 57]. En ce qui concerne la répartition hebdomadaire des cas de FHV de 2020 à 2022 dans la wilaya, les données indiquent que la moughataa de Kiffa (Fig. 2) a enregistré le plus grand nombre de cas en septembre et novembre, avec un pic hebdomadaire notable pendant le mois d’octobre de l’année 2020 (Fig. 3).

**Tableau I:** Évaluation des caractéristiques de la surveillance des FHV de 2020 à 2022 Assessment of FHV surveillance characteristics from 2020 to 2022

Caractéristiques	Moyenne (%)	IC95%
Utilité globale	51	[16- 85]
Simplicité	63	[33 - 94]
Flexibilité	46	[16- 77]
Acceptabilité	46	[21 - 70]
Réactivité	64	[39- 89]

**Tableau II:** Répartition des résultats des prélèvements de 2020 à 2022 selon la VPP Distribution of sample results from 2020 to 2022 based on PPV

Résultats des prélèvements	FVR		FHCC	
	Effectif (n)	Fréquence (%)	Effectif (n)	Fréquence (%)
Positif	24	28	0	0
Négatif	63	72	0	0
**Total**	**87**	**100**	**0**	**0**

## Discussion

Les résultats de cette étude ont indiqué certains points forts du système de surveillance FHV dans la wilaya de l’Assaba. Il s’agit notamment de l’utilité, de la simplicité et de la réactivité du système. Par contre, l’acceptabilité et la flexibilité doivent être améliorées. La notification a été régulière sur tous les sites de notification. Nous estimons que notre étude est représentative de la seule région (wilaya) de l’Assaba, car la totalité des personnels affectés à cette activité de surveillance dans la région a participé à cette enquête. Cependant, l’étude a également montré des lacunes majeures dans le bon fonctionnement de la surveillance des FHV à Assaba. Nous avons observé des problèmes de qualité et de validité des données avec les cas suspects de FHV ainsi que des problèmes concernant les ressources et la formation inadéquates du personnel. Les caractéristiques les plus faibles du système, telles que la qualité des données, pourraient être dues à une formation inadéquate des agents de surveillance sur la collecte et la gestion des données, ce qui entraîne le manque d’exhaustivité et de validité constaté dans cette évaluation. Cela pourrait à son tour être attribué en partie aux contraintes financières susmentionnées face aux priorités concurrentes si courantes dans les pays en voie de développement [2]. Les données générées par le système, issues de la détection et de la notification effectuées par des professionnels de la santé ou des établissements de santé, sont généralement fiables et elles sont conformes à des objectifs, des procédures et des contraintes spécifiques. L’évaluation que nous avons menée nous a permis d’analyser des données secondaires dont la qualité peut ne pas être optimale. Il est important de noter que les wilayas périphériques sont souvent plus vulnérables aux phénomènes émergents, avec un accès limité aux structures de soins et encore faible aux capacités diagnostiques spécialisées. Une étude similaire au Ghana et une autre en Guinée sur la surveillance de la fièvre à virus Ebola ont également montré que la mauvaise qualité des données, la disponibilité et la validité des données étaient des problèmes majeurs qui avaient contribué à la grande épidémie d’Ebola en Afrique de l’Ouest [1, 23]. La réponse de la santé publique aux épidémies est affectée par l’exhaustivité et l’exactitude des informations disponibles [1]. Plus tôt les cas sont décelés, plus il est probable qu’une intervention empêche l’apparition d’autres cas, surtout si elle a lieu avant la phase de croissance logarithmique de l’épidémie, d’où l’importance d’un système de surveillance performant.

La promotion de l’élevage familial favorise un contact étroit entre les animaux et les populations humaines, augmentant ainsi le risque de transmission de maladies zoonotiques [14]. Il est donc nécessaire de mettre en œuvre des moyens d’information de la population, du personnel médical, vétérinaire et les agents de l’environnement dans les zones endémiques et enzootiques, sur la maladie et sur les moyens de prévention. L’application de l’approche « Une seule santé », qui encourage la collaboration synergique de multiples disciplines en vue d’optimiser la santé des individus, des animaux et de l’environnement, n’est pas toujours systématique dans des contextes similaires.

## Recommandations

À la suite de cette étude, plusieurs suggestions émergent pour améliorer la gestion des FHV dans la wilaya d’Assaba. À la DRS, il est recommandé de superviser régulièrement les activités de surveillance dans les moughataas, d’organiser des formations sanitaires trimestrielles. Pour le Centre hospitalier de Kiffa, il est conseillé de renforcer la collaboration avec la DRS et l’INRSP pour la confirmation des cas suspects de FHV chez les patients hospitalisés. Enfin, les responsables des formations sanitaires de la région sont encouragés à intégrer la surveillance des FHV en tant qu’activité permanente pour assurer une détection précoce des cas, avec une notification immédiate des cas suspects au niveau supérieur.

## Contribution des auteurs

Boushab Mohamed BOUSHAB : revue de littérature, rédaction du manuscrit.

Pauline Kiswendsida YANOGO, Pauline Kiswendsida YANOGO, Mohamedou Hmeied MAHAM, Herman YODA, Djibril BARRY, Ahmed El-BARA, Nicolas MEDA : apport critique, correction du manuscrit et approbation de la version finale à publier.

## Déclaration de liens d’intérêts

Les auteurs déclarent ne pas avoir de liens d’intérêt.
